# Manipulating the Spin State of Perovskite Cs_3_Bi_2_Br_9_ by Co‐Doped for Efficient Photocatalytic CO_2_ Reduction

**DOI:** 10.1002/advs.202511617

**Published:** 2025-11-20

**Authors:** Fahui Wang, Yongmei Xia, Zuming He, Gang He, Juan Zhang, Jiangbin Su, Guihua Chen, Xiaofei Fu, Muhammad Saboor Siddique, Yu Xie, Guoliang Dai

**Affiliations:** ^1^ Jiangxi Provincial Key Laboratory of Power Batteries & Energy Storage Materials Xinyu University Xinyu 338004 China; ^2^ School of New Energy Science & Engineering Xinyu University Xinyu Jiangxi 338004 P. R. China; ^3^ School of Resources and Environmental Engineering Jiangsu University of Technology Changzhou 213001 P. R. China; ^4^ School of Microelectronics and Control Engineering Changzhou University Changzhou 213164 P. R. China; ^5^ School of Pharmaceutical and Chemical Engineering Taizhou University Jiaojiang 318000 P. R. China; ^6^ Institute of Environment and Ecology Tsinghua‐Shenzhen International Graduate School Tsinghua University Shenzhen 518055 China; ^7^ School of Environmental and Chemical Engineering Nanchang Hangkong University Nanchan 330063 P. R. China; ^8^ School of Chemistry and Life Sciences Suzhou University of Science and Technology Suzhou 215009 P. R. China

**Keywords:** Co doping, CO_2_ reduction, perovskite photocatalysis, spin polarization, Zeeman effect

## Abstract

The photocatalytic conversion of CO_2_ into the renewable fuels is a promising strategy to address energy and environmental challenges, however, its limited application is mainly attributed to the inefficient charge separation and lack of active sites in conventional catalysts. Here, a spin‐polarization strategy using Co^2^⁺ doping in lead‐free perovskite Cs_3_Bi_2_Br_9_ (CBB) synergized with an external magnetic field (MF), is reported to achieve highly efficient CO_2_ reduction. The optimized Co‐doped CBB (0.2CBB) exhibited a 2.6‐fold enhancement in CO production rate (35.04 µmolg^−1^h^−1^) compared to the pristine CBB, with further improvement to 86.56 µmolg^−1^h^−1^ under 200 mT MF. Advanced characterizations together with the density functional theory calculations further revealed that the Co doping introduces spin‐polarized electrons, suppresses charge recombination, and elongates the carrier lifetime (6.68 ns vs 5.20 ns in CBB). The Zeeman effect under MF activates the additional spin‐polarized carriers, while the Co sites lower the energy barrier for ^*^COOH intermediate formation (ΔG = −0.59 vs −0.38 eV in CBB), as confirmed by the in situ FT‐IR and Gibbs free energy analysis. This work pioneers the integration of spin manipulation and MF‐assisted catalysis in perovskites, offering a novel pathway for the design of high‐performance photocatalytic systems.

## Graphical abstract

The photocatalytic conversion of CO_2_ into the renewable fuels is a promising strategy to address energy and environmental challenges. Here, we reported a spin‐polarization strategy using Co^2+^ doping in lead‐free perovskite Cs_3_Bi_2_Br_9_ synergized, to achieve highly efficient CO_2_ reduction. The optimized Co‐doped CBB exhibited a 2.6‐fold enhancement in CO production rate compared to the pristine CBB. Advanced characterizations and density functional theory calculations revealed that the Co doping introduces spin‐polarized electrons, suppresses charge recombination, and elongates the carrier lifetime, while the Co sites lower the energy barrier for *COOH intermediate formation.

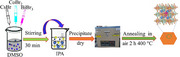

## 1 Introduction

With the rapid development of society and the economy, the excessive consumption of fossil fuels and the massive emission of carbon dioxide (CO_2_) lead to severe energy and environmental issues, such as the greenhouse effect, rising global sea levels, and extreme weather events.^[^ ^1^ ^]^ To cope with these challenges, the photocatalytic conversion of CO_2_ into renewable fuels (e.g., CH_4_, CO, and various hydrocarbons) has emerged as a promising solution to mitigate the global climate crisis.^[^ ^2^ ^]^ In an efficient photocatalytic system converting CO_2_ into renewable fuels, semiconductor photocatalysts play a critical role. These photocatalysts generally possess a relatively negative conduction band position, a broad range of sunlight absorption, and high mobility and separation efficiency of photogenerated charge carriers. However, the design of photocatalysts with high efficiency and stability is still a significant challenge among researchers. Therefore, a stable, ecofriendly, and solar‐driven semiconductor‐based photocatalyst is considered one of the most effective approaches to deal single‐handedly with both aspects of energy and environmental crisis.

In recent years, metal halide perovskites have gained significant attention as green photocatalysts in the field of photocatalysis owing to their long carrier lifetime, high carrier mobility, and tunable bandgap.^[^ ^3^ ^]^ Among these photocatalysts, Cs_3_Bi_2_Br_9_ (CBB) is a nontoxic lead‐free perovskite having stable chemical properties together with a sufficient negative conduction band (CB) potential, making it a promising candidate for an effective CO_2_ reduction due to its strong reducing power. However, pristine CBB demonstrates poor CO_2_ photoreduction performance because of the rapid recombination of photogenerated carriers and the lack of active sites.^[^ ^4^ ^]^ Furthermore, the band structure and photoelectrical properties of the metal halide perovskites are primarily determined by the MX_6_ octahedral unit (where M represents a divalent or trivalent metal cation, and X represents a halide anion), where the electronic structure is mainly contributed by the *M‐s* and *X‐p* orbitals.^[^ ^5^ ^]^ The configuration and modulation of the metal cations provide a feasible approach to regulating the band structure and photoelectrical properties, thereby enhancing photocatalytic activity. To address the limitations of CBB and improve its photocatalytic performance, numerous strategies have been explored, for instance, Ren et al. constructed an S‐scheme heterojunction of Cs_3_Bi_2_Br_9_/BiOBr through in situ partial conversion to enhance photocatalytic N_2_ fixation.^[^ ^6^ ^]^ Similarly, Xia and colleagues developed Cs_3_Bi_2_Br_9_/FeS_2_ Z‐scheme charge transfer systems, where the photoinduced self‐heating effect synergistically improved the solar‐driven photocatalytic H_2_ production.^[^ ^7^ ^]^ Moreover, Gao's research team fabricated the heterojunction interfaces by in situ growth of CBB on ultrathin BiVO_4_ nanosheets to boost the photocatalytic activity.^[^ ^8^ ^]^ Despite these advancements, the efficiency of the photocatalytic activity remains unsatisfactory due to inherent material defects.

Recently, modifying the composition and structure of the CBB has been emerged as an effective strategy to enhance the surface charge separation and improve photocatalytic efficiency.^[^ ^9^ ^]^ For example, Kim et al reported that the incorporation of Fe ions into CsPbBr_3_ not only expands the light absorption range and prolongs the lifetime of photogenerated electrons and holes, but also promotes the charge separation, thereby enhancing the stability and hydrogen evolution performance.^[^ ^10^ ^]^ Likewise, Yue et al. synthesized the defect‐rich CBB using a photoinduction strategy, which introduced the abundant surface defects to facilitate the surface charge separation and improve photocatalytic hydrogen production efficiency. Moreover, spin, as an inherent characteristic of electrons, has been identified as a key factor in enhancing photocatalyst performance.^[^ ^11^ ^]^ In this aspect, spin manipulation to suppress the recombination of photoexcited electrons has been recognized as a promising strategy to improve the photo‐ and electrocatalytic reactions.^[^ ^12^ ^]^ For instance, Guo's research team utilized a one‐pot hot injection method to prepare Mn^2+^‐doped Cs_3_Cu_2_Br_5_, achieving an efficient electron‐hole separation together with the extended charge carrier lifetime. Under an external magnetic field (MF), spin‐polarized electrons reduced the charge recombination and increased the availability of electrons and holes for surface redox reactions, enhancing photocatalytic performance.^[^ ^13^ ^]^ Similarly, Xia et al. demonstrate the Ni^2+^ doping‐induced spin‐polarized band splitting in Ni‐BaTiO_3_ nanofibers under photoexcitation, generating spin‐excited electrons and holes, which further inhibited the photogenerated charges recombination and prolonged their lifetime for CO_2_ photoreduction.^[^ ^14^ ^]^ These findings further highlight the potential of spin manipulation in improving the efficiency of photocatalytic CO_2_ reduction processes.^[^ ^15^ ^]^

Inspired by the successful enhancement of photocatalytic activity through spin manipulation, we propose an integrated strategy to polarize the spin of photoexcited electrons and leverage spin polarization to induce CO_2_ reduction. In this aspect, Cobalt (Co) ions are considered as the promising candidates for that specific purpose. The magnetic properties of the Co dopants can induce spin polarization, suppress the recombination of photoexcited electrons and holes, and simultaneously reduce the adsorption‐free energy of intermediates in the CO_2_ reduction process on CBB, thereby enhancing CO_2_ adsorption on the catalyst surface. Moreover, the aforementioned spin manipulation under the action of applied MF, further improves the reduction efficiency of CO_2_, attributing to the Zeeman effect. However, the effects of spin manipulation in cobalt‐doped CBB and its impact on CO_2_ reduction are yet to be explored.

Herein, we report the synthesis of Co‐doped CBB catalysts by introducing Co^2^⁺ ions into CBB nanosheets through an antisolvent precipitation and annealing process. These catalysts were employed for the photocatalytic reduction of CO_2_, selectively producing CO and CH_4_, to improve photocatalytic performance and structural stability. The photocatalytic CO_2_‐to‐CO conversion efficiency was significantly enhanced due to the successful incorporation of Co dopants, because of the induced spin‐polarized electrons, inhibited charge recombination, and extended charge carrier lifetime. Furthermore, upon an external MF application, the Zeeman effect activated the additional spin‐polarized carriers and active sites, further boosting the photocatalytic performance of Co‐doped CBB. To comprehensively investigate the charge kinetics and photocatalytic mechanisms, we employed a range of advanced characterization techniques, including magnetic circular dichroism spectroscopy (MCD), in situ steady‐state and transient photoluminescence (PL) spectra, in situ transient photocurrent measurements, in situ electrochemical impedance spectroscopy, and in situ electron paramagnetic resonance (EPR) spectra. Additionally, density functional theory (DFT) calculations and in situ Fourier transform infrared spectroscopy (FT‐IR) were utilized to provide deeper insights into the reaction pathways. This study not only facilitates the conversion of CO_2_ into high‐value‐added chemicals but also holds a significant potential to address the global energy and environmental challenges.

## 2 Materials and Methods

### 2.1 Materials

The specific details of all the analytical grade (AR) chemicals and reagents, which were used directly without any further purification in this study, unless otherwise stated, are reported in Section S1 (Supporting Information). Deionized water was utilized for all experimental procedures.

### 2.2 Preparation of Cs_3_Bi_2‐x_Co_x_Br_9_ Nanosheets

The Cs_3_Bi_2‐x_Co_x_Br_9_ nanosheets were synthesized via ions doping precipitation and annealed method, synthetic procedure: Typically, 3 mmol of CsBr, 2‐*x* mmol of BiBr_3_ and different amounts *x* mmol of CoBr_2_ (i.e., *x* = 0, 0.1, 0.2, 0.3 mmol) were dissolved in a 10 mL of dimethyl sulfoxide (DMSO), following continuous stirring for 30 min. Afterward, the above precursor was quickly transferred into the isopropanol (IPA, 50 mL) under vigorous stirring. Subsequently, the reaction mixture was allowed for 5 min, and then the obtained yellow mixture of Cs_3_Bi_2‐_ *_x_* Co*_x_* Br_9_ was centrifuged at 4000 rpm for 3 min to remove large particles, washed with ethanol and the obtained nanosheets were dried in a vacuum oven at 80 °C for 24 h. Lastly, the Cs_3_Bi_2‐_ *_x_* Co*_x_* Br_9_ nanosheets were annealed at 400 °C with a 5 °C min^−1^ heating rate in the vacuum tube‐type furnace for 3 h. The obtained Cs_3_Bi_2_Br_9,_ Cs_3_Bi_1.9_Co_0.1_Br_9,_ Cs_3_Bi_1.8_Co_0.2_Br_9_ Cs_3_Bi_1.7_Co_0.3_Br_9,_ nanosheets were labeled as CBB, 0.1CBB, 0.2CBB, 0.3CBB, respectively.

### 2.3 Characterization

Additional procedures to evaluate the prepared samples, including material characterization, photoelectrochemical tests, MCD, EPR tests, DFT, and In situ FT‐IR, are given in Sections S2–S7 (Supporting Information).

### 2.4 Photocatalytic CO_2_ Reduction

The photocatalytic CO_2_ reduction was performed in a 50 mL electromagnet quartz reactor with tunable MF strength above and below a custom seal (that is, to change the MF intensity by controlling the distance). A 300 W Xe lamp with a UV cutoff filter (> 380 nm) provided irradiation, and the temperature was stabilized using a water circulation system without external heating. Prior to illumination, 10 mg of photocatalyst was dispersed onto the base of the reactor, followed by the addition of 10 µL of deionized water. The system was then purged with CO_2_ for 30 min at a flow rate of 15 mL min^−1^ to thoroughly eliminate air from the reactor. Upon light exposure, gas samples (500 µL/h^−1^) were collected and analyzed via gas chromatography (GC9700, Fuli Analytical Instrument Co., Ltd.).

## 3 Results and Discussion

### 3.1 Structure and Morphology

**Figure 1:**
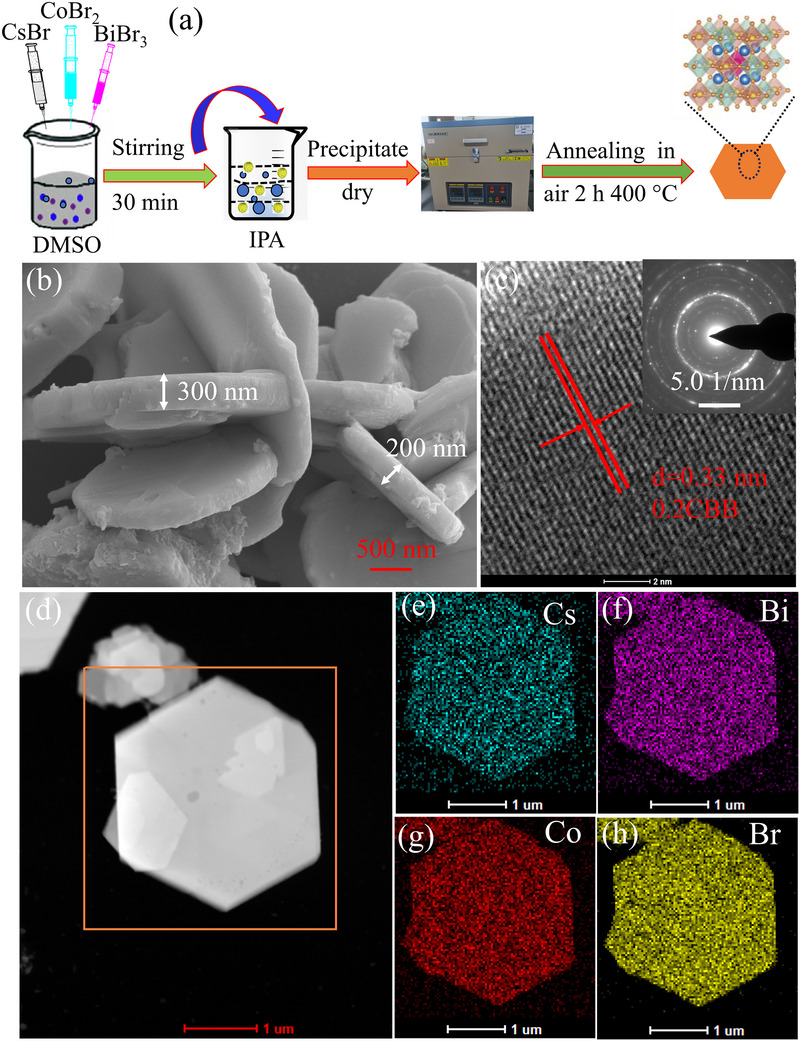
a) The synthesis route of Co‐doping CBB nanosheets; b) SEM image of CBB, c) HRTEM and SAED (inset) images; d) HAADF‐STEM, and e–h) EDX elemental mapping images of 0.2CBB nanosheets.

The Co‐doped CBB nanosheets were fabricated with the ions doping method and calcination technique (**Figure** **1**a). The morphologies and microstructures of the as‐synthesized samples were characterized by scanning electron microscopy (SEM), transmission electron microscope (TEM), and high‐resolution TEM (HR‐TEM). The pure CBB showed a typical nanosheet‐type structure with an approximate thickness of 200–300 nm, width is ≈1– 2 µm (Figure 1b). Whereas the incorporation of the magnetic Co into the perovskite CBB semiconductors indicates the original intact morphology similar to that observed for the pristine CBB (Figure S1, Supporting Information), attributing to the size match between Co^2+^ (0.97 Å) and Bi^+^ (1.03 Å) makes the Co ions more likely to replace Bi rather than Cs^+^(1.63 Å) ions, thus maintaining the structural stability of the lattice by avoiding lattice overstrain strain and distortion.^[^ ^16^, ^17^, ^18^ ^]^ Moreover, the TEM (Figure S2, Supporting Information) and HR‐TEM (Figure 1c) images of 0.2CBB distinctly visualize the characteristic lattice spacing of 0.33 nm, which corresponds to the (002) plane of perovskite structures CBB (JCPDS No. 44‐0714), indicating the unchanged morphological structure of the CBB even upon Co‐doping.^[^ ^19^ ^]^ Furthermore, the selected area electron diffraction (SAED) pattern presents few diffraction rings (in the inset of Figure 1c), suggesting the polycrystalline nature of the catalyst. Additionally, the high‐angle annular dark field scanning transmission electron microscopy (HAADF‐STEM), and corresponding energy‐dispersive X‐ray (EDX) spectroscopy mapping images and spectrum (Figure 1d–h; Figure S3, Supporting Information), also proved the coexistence and homogeneous distribution of Cs, Bi, Co, and Br in 0.2CBB, further demonstrating the successful synthesis of 0.2CBB. These above findings were also found to be consistent with the inductively coupled plasma atomic emission spectroscopy (ICP‐AES, Table S1, Supporting Information), which further correlates with that of the XRD and XPS.

**Figure 2:**
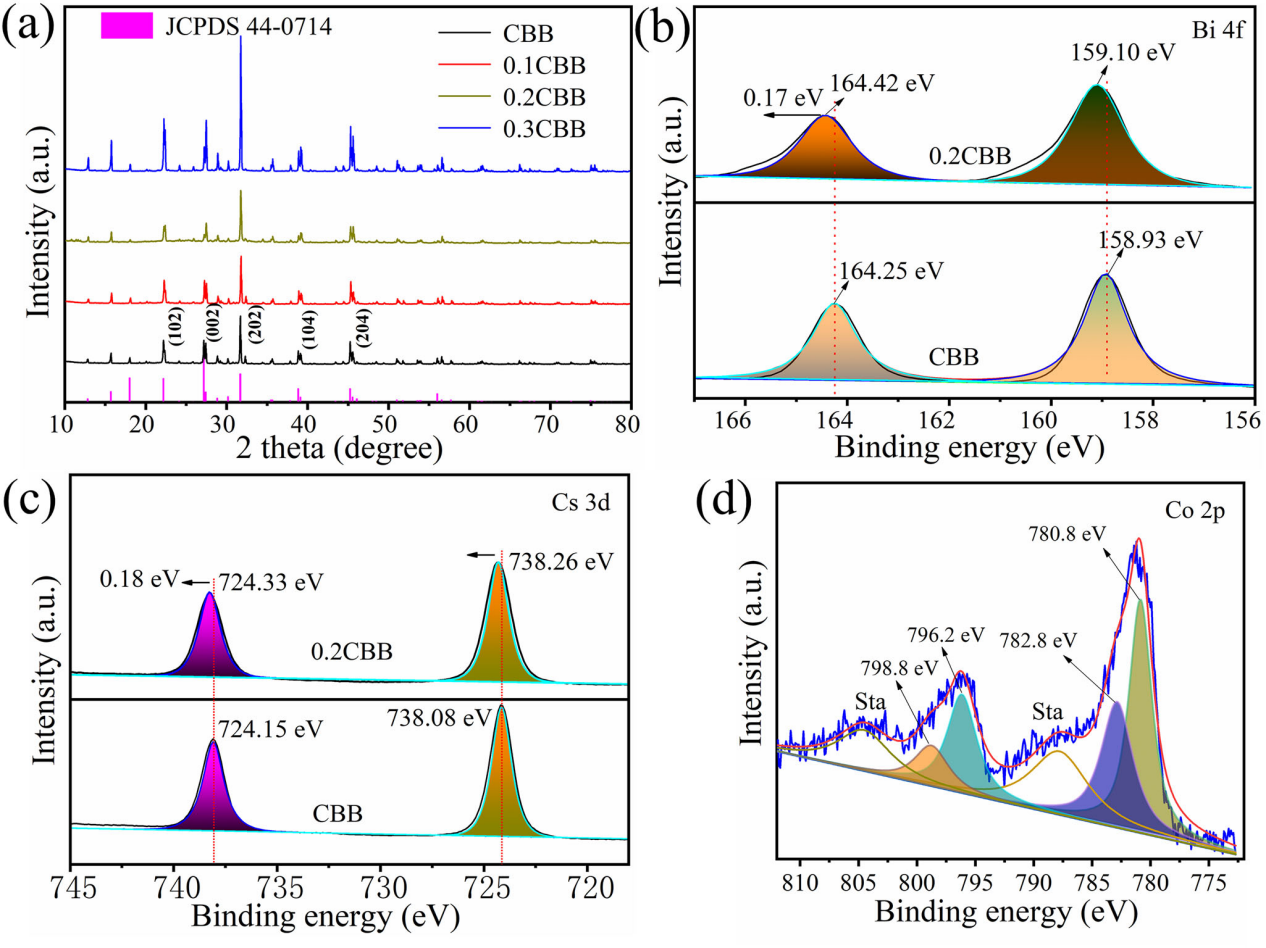
a) XRD patterns of pure CBB and Co‐doped CBB nanosheets; High‐resolution XPS spectra b) Bi 4f, c) Cs 3d of CBB and 0.2CBB; d) Co 2p of 0.2CBB.

The crystalline structure of the as‐prepared samples was also analyzed via X‐ray diffraction (XRD), the sharp diffraction peaks located at 21.19, 27.16, 31.68, 38.83, and 45.24° correlate well with the (102), (003), (202), (104) and (204) planes of hexagonal phase CBB (JCPDS No. 44‐0714) (**Figure** **2**a), respectively, and no impurity peaks were observed.^[^ ^20^ ^]^ Noticeably, after the introduction of Co^2+^, the positions of the diffraction peaks slightly shifted toward the higher angles (Figure S4, Supporting Information), confirming the lattice distortion. This shift was mainly attributed to the smaller ionic radius of the Co^2+^ (0.97 Å) as compared to that of the Bi^3+^ ions (1.03 Å), indicating the incorporation of Co atoms into the lattice of CBB, as already confirmed by the HAADF‐TEM equipped with EDX element mapping. Moreover, the chemical valence states of the CBB and 0.2CBB were investigated by the XPS measurements. The XPS spectra of the CBB and 0.2CBB showed the presence of Cs, Bi, Co, and Br in the samples (Figure S5, Supporting Information), as consistent with the EDX and XRD results. Figure 2b displayed the Bi 4f peaks in the pure CBB located at 158.93 and 164.25 eV, corresponding to the Bi 4f_7/2_ and 4f_5/2_, respectively.^[^ ^21^ ^]^ However, the high‐resolution Cs 3d spectra of CBB and 0.2CBB can be convolved into two peaks at 724.5 and 738.08 eV, credited to the Cs 3d_5/2_ and 3d_3/2_, respectively (Figure 2c).^[^ ^8^, ^12^ ^]^ Whereas, the Br 3d_5/2_ and Br 3d_3/2_ peaks of CBB at 68.1 and 69.1 eV (Figure S6, Supporting Information).^[^ ^22^ ^]^ Additionally, the Co 2p spectra deconvoluted and fitted peaks resulted in four peaks at binding energies of 780.8, 782.8, 796.2, and 798.8 eV for 0.2CBB, which can be attributed to the Co^3+^ 2p_3/2_, Co^2+^ 2p_3/2_, Co^3+^ 2p_1/2_, and Co^2+^ 2p_1/2_, respectively (Figure 2d), implying the coexistence of Co^2+^ and Co^3+^,^[^ ^23^ ^]^ while a satellite peak in Co‐Br reflects the +3 oxidation state. Noticeably, the Br 3d, Cs 3d, and Bi 4f peaks were shifted slightly toward the higher binding energies in 0.2CCB, indicating the enhancement of chemical interactions between Co and Br, which mainly contributes to the improved stability of 0.2CBB.

### 3.2 Optical Properties and Band‐Gap Analysis

**Figure 3:**
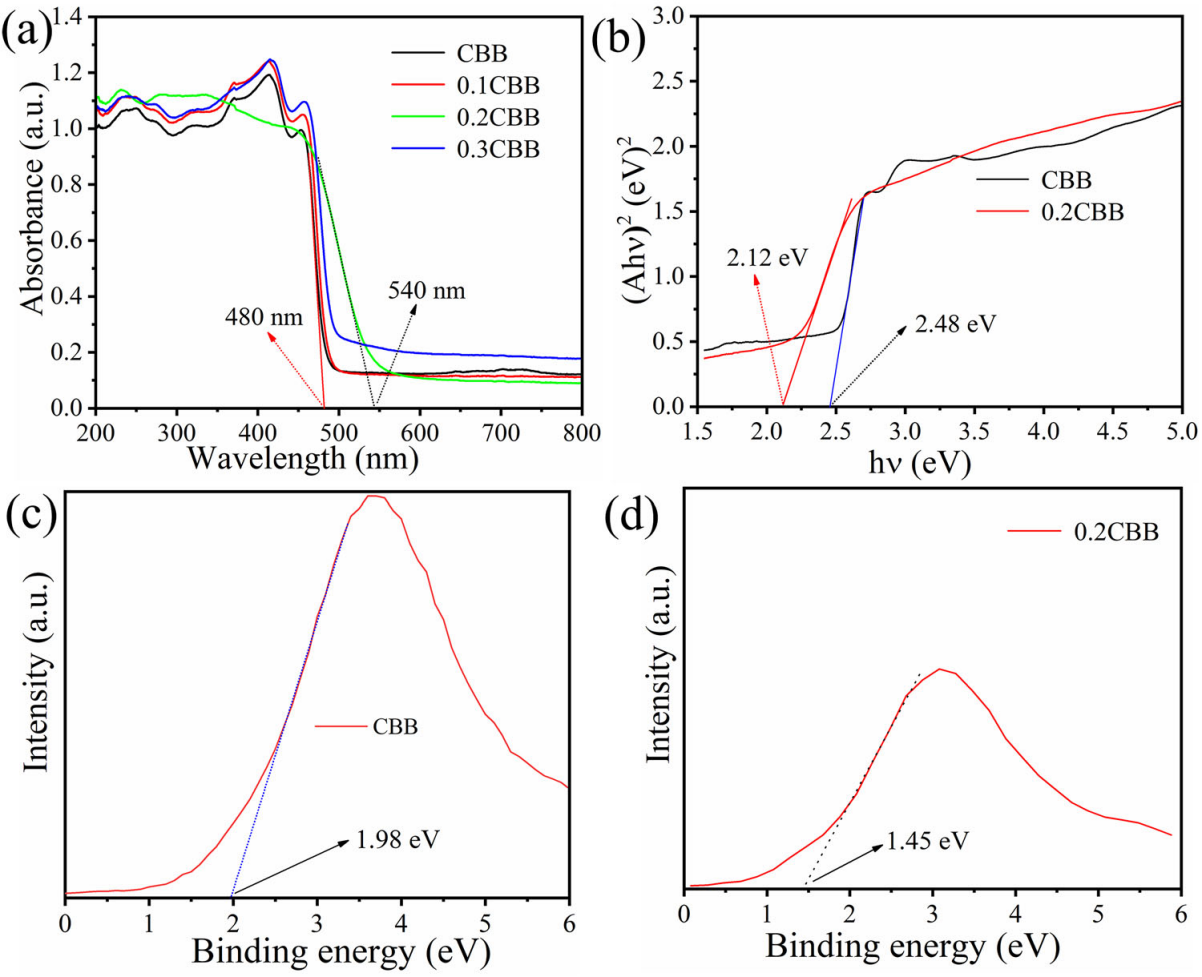
a) UV–vis DRS spectra and physical photographs are shown in the inset, b) Kubelka–Munk plots converted from the UV–vis DRS spectra; c,d) XPS valence band spectra.

It is generally believed that the optical absorption efficiency of the semiconductors plays a vital role in photocatalytic activity.^[^ ^24^ ^]^ For the pristine CBB, the UV–vis absorption was observed ≈480 nm, however, the introduction of Co can significantly broaden the light response range of CBB, because of the possible interaction between Br and Co atoms (**Figure** **3**a). This change is advantageous as it enhances the light absorption capacity of CBB. Moreover, the energy band structures were further evaluated using Kubelka‐Munk plots and XPS valence band (VB) spectra to obtain the band gap (Eg) and VB positions of CBB 2.48 and 1.98 eV, respectively (Figure 3b,c). Similarly for 0.2CBB, the band gap (Eg) and VB were 2.12 and 1.45 eV, respectively (Figure 3b,d). In addition, the CB positions of CBB and 0.2CBB were found to be −0.5 and −0.67 eV, respectively, as calculated by using E_CB_ = E_VB_‐Eg, and these findings have also been verified by Mott–Schottky plots (Figure S7, Supporting Information). Based on the above experimental results, the energy band structure of CBB and doped 0.2CBB are shown in Figure S8 (Supporting Information).

### 3.3 Photocatalytic CO_2_ Reduction

**Figure 4:**
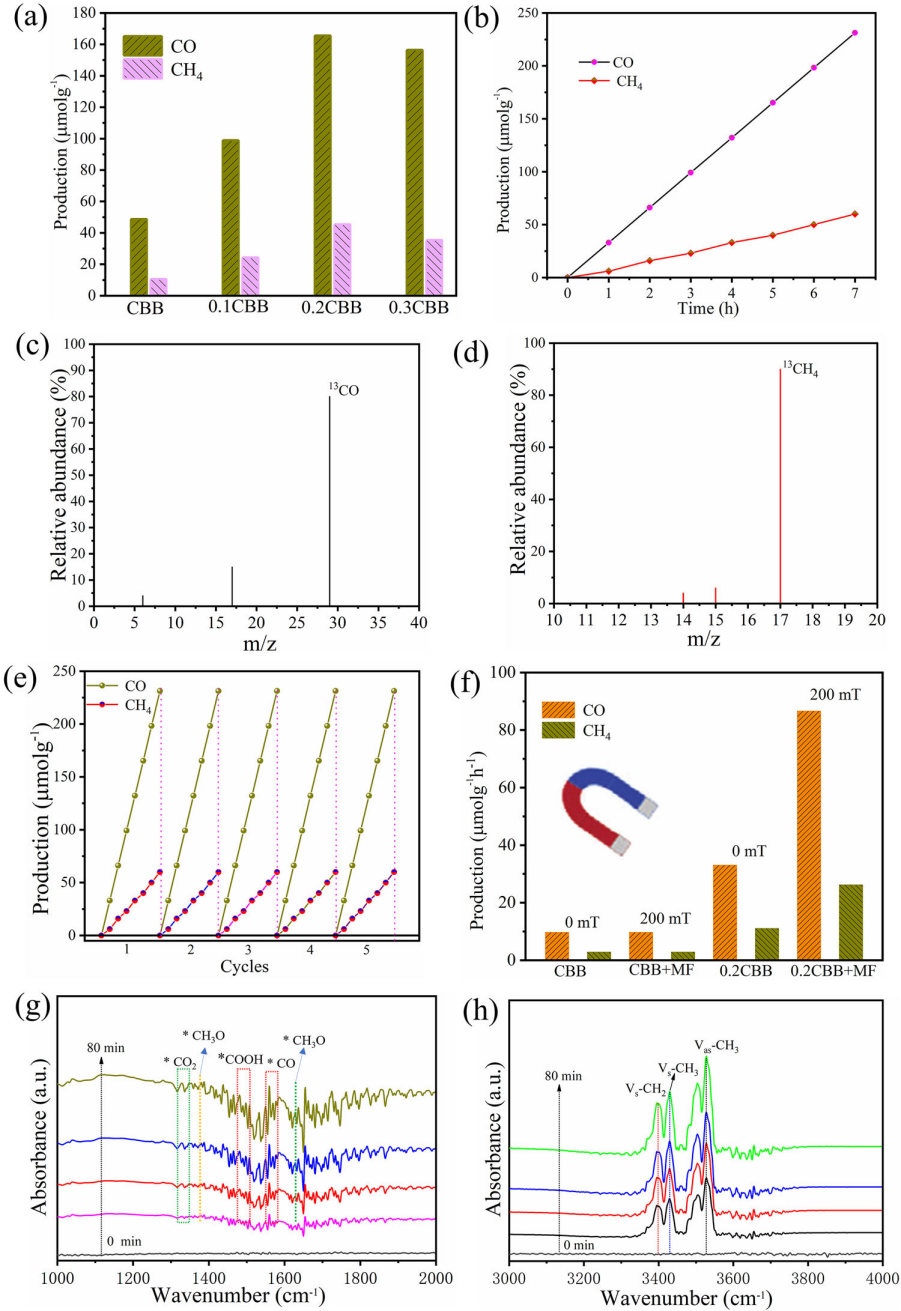
a) Photocatalytic yield of CH_4_ and CO in five hours of pure CBB and Co‐doped CBB; b) Time‐dependent production of CH_4_ and CO of 0.2CBB; Mass spectra of the products of c) CO and d) CH_4_ with isotope labeling using ^13^CO_2_. e) Cycling photocatalysis performance of 0.2CBB; f) Photocatalytic CO_2_ reduction performance under the MF: g,h) in situ FT‐IR spectrum of photocatalytic CO_2_ reduction under the different illumination time of the 0.2CBB photocatalyst.

Encouraged by the above results, the photocatalytic CO_2_ reduction performance was conducted in a reactor equipped with a MF. A 300 W Xe lamp equipped with a UV cut‐off filter (> 380 nm) was used as a visible‐light source, and the gaseous products were detected by gas chromatography. After 5 h of illumination, the photocatalytic yields of CH_4_ (13.6 µmolg^−1^) and CO (49.3 µmolg^−1^), reduced the photocatalytic performance of the pristine CBB, due to rapid charge recombination (**Figure** **4**a). It is also noteworthy that CO and CH_4_ production was significantly enhanced upon introducing Co into the perovskite CBB nanosheets. Especially, the yields of CO and CH_4_ reached the maximum potential of 35.04 and 10.21 µmolg^−1^h^−1^, for 0.2CBB owing to the efficient charge separation of perovskite nanodots, respectively. However, the further increase of Co doping in perovskite CBB nanosheets slightly decrease the production rate of CO and CH_4_, attributing to the photo‐excited charge carriers trapped into the defects induced by Co dopant and recombine through the defect‐assisted non‐radiative recombination. Extremely high average production rates and selectivity for CO were obtained at 0.2CBB, i.e., 35.04 µmolg^−1^h^−1^, and 79.8%, respectively (Figure 4b). Compared with other photocatalysts, 0.2CBB simultaneously achieved an excellent CO selectivity and yield (Table S2). To clarify the origin of CO and CH_4_, the ^13^CO_2_ and ^17^CH_4_ isotopic labeling experiments were performed using gas chromatography‐mass spectrometry (GC‐MS). The GC‐MS findings further depicted that the significant peaks with *m*/*z* of 29 (assigned to ^13^CO) and 17 (assigned to ^13^CH_4_) were identified (Figure 4c,d), which suggests that the origin of the carbon product obtained is related to CO_2_.^[^ ^25^ ^]^ Moreover, under the identical conditions, a series of control experiments were conducted without the presence of light, catalyst, and replacing CO_2_ with N_2_, the carbonaceous products were also not detected, revealing that CO_2_ was the only carbon source for the reduction products (Figure S9, Supporting Information). Additionally, the photocatalytic cyclability and stability of 0.2CBB were also tested, and a negligible decline in the production yields of CH_4_ and CO was observed even after 5 cycles (Figure 4e). Importantly, XRD (Figure S10a, Supporting Information), SEM (Figure S10b,Supporting Information) and XPS (Figure S10c, Supporting Information) of 0.2CBB still maintained their structural integrity both macroscopically and microscopically, indicating the excellent photostability of the 0.2CBB nanosheets. Next, we applied the MF of 200 mT, the 0.2CBB showed the CO and CH_4_ rate of 86.56 and 25.36 µmol g^−1^h^−1^, which was 2.62 and 2.38 times higher than that of 0.2CBB without using the MF, respectively (Figure 4f). However, in the pristine CBB, a negligible yield difference was observed for both with and without the MF application, confirming the Co doping‐based enhanced ferromagnetism in CBB.

Next, the in situ FT‐IR analysis was conducted to trace the dynamical evolution of reaction intermediates, which further confirmed the gradual enhancement of the peaks of key intermediates upon prolonged exposure to light irradiation, the peaks at 1390 cm^−1^ can be assigned to ^*^CO_2_ (Figure 4g,h). The infrared peaks at ≈1430 and 1743 cm^−1^ were observed in the spectra, both corresponding to ^*^CH_3_O.^[^ ^26^ ^]^ Moreover, the ^*^COOH intermediate was observed at 1549 cm^−1^, which was found to be a crucial intermediate for CO_2_ reduction to CO.^[^ ^27^ ^]^ Additionally, the signal peak of the chemisorption ^*^CO species at ≈1652 cm^−1^ also supports the CO as the main product during CO_2_ reduction. In addition, the peaks at 3400, 3416, and 3520 cm^−1^ were assigned to the C‐H symmetric stretching vibration bands of methylene, which can further enhance the efficient photocatalytic CO_2_ reduction into CH_4_.^[^ ^28^ ^]^

### 3.4 Exploring the Mechanism of Photocatalytic Enhancement

**Figure 5:**
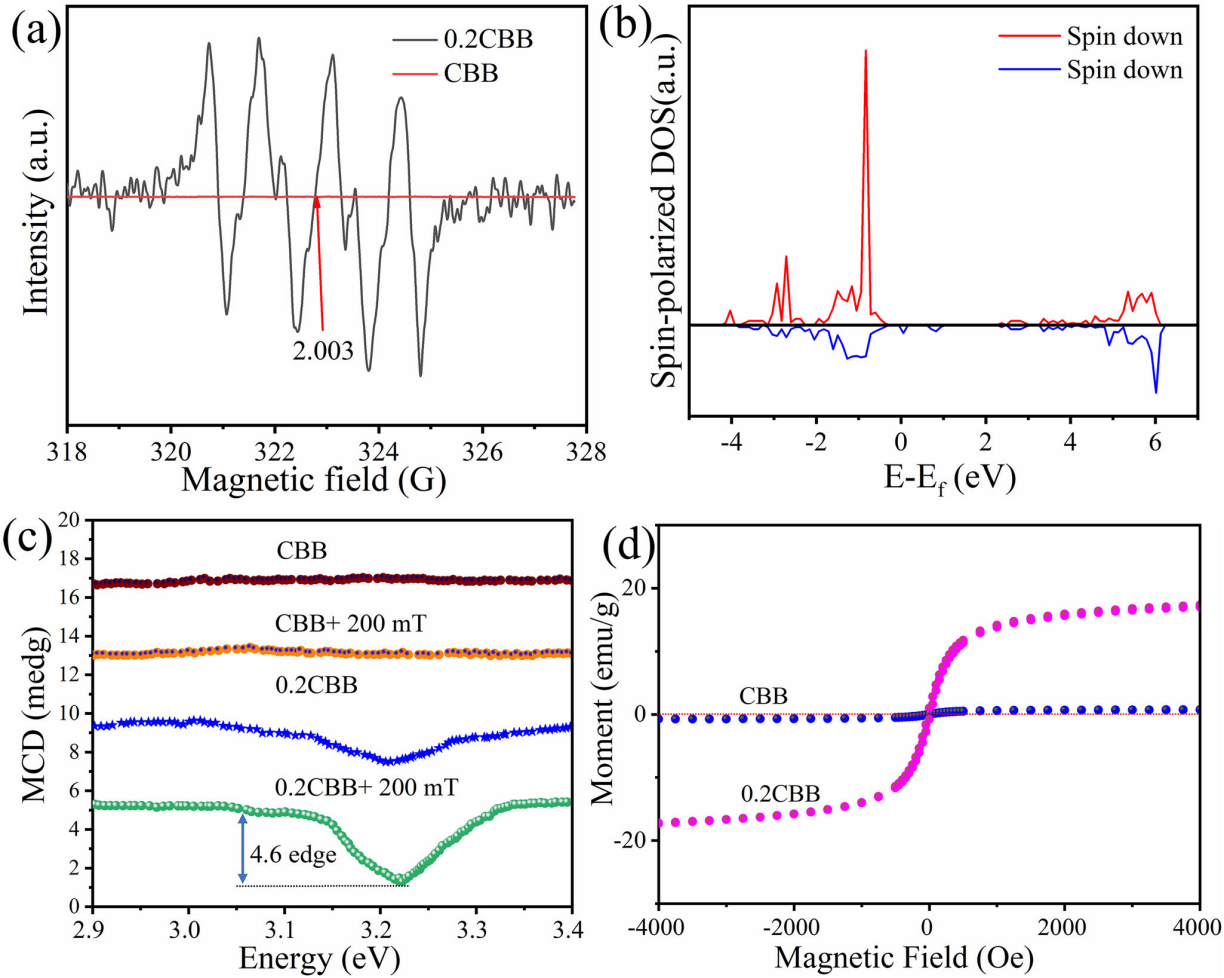
a) EPR spectra, b) the spin‐polarized DOS, c) MCD, and d) the Magnetic hysteresis loop of CBB and 0.2CBB.

In general, the MF‐induced Zeeman effect can regulate the spin polarization of nanostructures, thus affecting the lifetime of the electron‐hole pairs.^[^ ^29^ ^]^ Therefore, we proposed that the Zeeman effect promoted the electric charge spin‐polarization, enabling efficient photocatalytic CO_2_ reduction. In this aspect, the EPR spectra of bare CBB showed no signal peaks as compared to the 0.2CBB (**Figure** **5**a). However, the 0.2CBB exhibits six sharp nuclear electron hyperfine splitting peaks under 200 mT (corresponding to a g‐2.003), exhibiting a significant Zeeman splitting phenomenon with well‐resolved hyperfine structures. This might originate from the unpaired electron spin in the 3d‐orbital of Co^2+^, thereby changing the local crystal structures of the CBB. Moreover, we also perform the DFT calculations for the spin polarization density of states (PDOS) of both bare CBB and 0.2CBB crystals and the difference in the DOS of the spin‐up and spin‐down electrons around the Fermi level. There is no spin polarization DOS observed in the bare CBB crystals (Figure S11, Supporting Information), indicating the absence of spin‐polarized electrons. In contrast, the spin‐polarized DOS (Figure 5b) exhibited a higher degree of spin polarization and more spin‐up electrons, further revealing that the Co doping introduces more spin‐up electrons near the Fermi level compared to CBB, disclosing its superior charge separation. According to the current theory, when linearly polarized light passes through the medium, the resonance absorption difference between left‐circularly polarized light and right‐circularly polarized light splits the energy under the magnetic circular dichroism MCD Zeeman MF (200 mT).^[^ ^30^ ^]^ Therefore, the Zeeman effect was further investigated by MCD spectra under an external MF, where the response of the external MF was found to be negligible toward the activity of pure CBB (Figure 5c). However, a significant MCD signal appeared against the 0.2CBB in the absence of MF, confirming the Co^2+^ doping‐induced construction of the spin‐polarized bands. In addition, upon increasing the MF intensity to 200 mT, a significant MCD signal difference (≈4.6 mdge) appeared for the 0.2CBB, indicating that Co^2+^ doping causes CBB vulnerable to spin‐polarized band splitting caused by the Zeeman effect of the MF. Further, we also studied the magnetization versus MF (M–H) measurements of 0.2CBB and CBB by conducting a vibrating sample magnetometer (VSM) at room temperature (Figure 5d). Compared with the pure CBB, 0.2CBB displayed para‐magnetism with a magnetization of 18.35 × 10^−3^ emu g^−1^ under 4 KOe, indicating the existence of spin‐polarized electrons for 0.2CBB.

**Figure 6:**
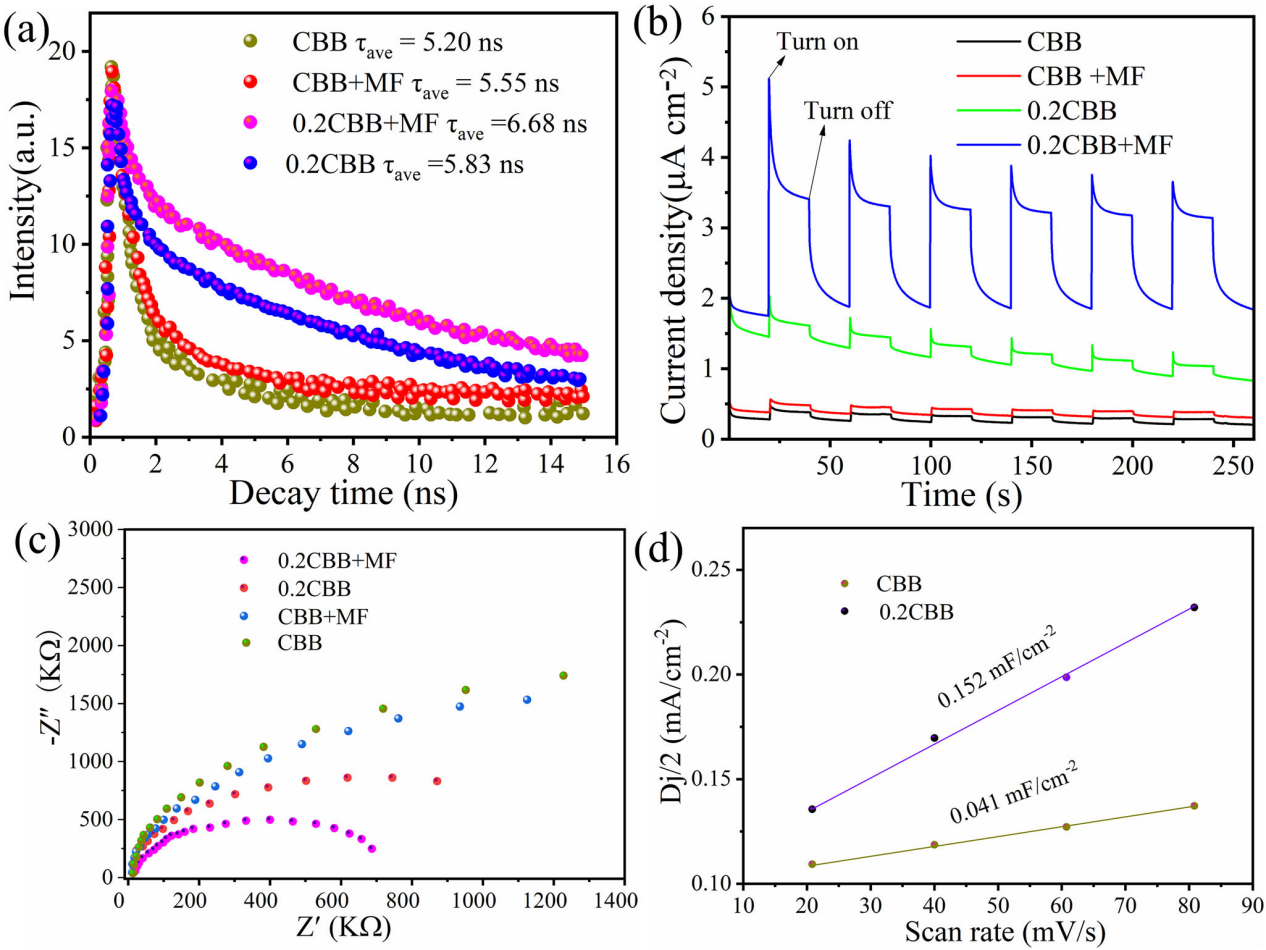
a) TR‐PL spectra, b) Transient‐state transient photocurrent densities, and c) Nyquist plots of EIS spectra of CBB and 0.2CBB with and without external MF (200 mT), respectively; d) The C_dl_ of CBB and 0.2CBB.

To unveil the photoexcited carrier dynamics under the Zeeman effect, we further performed the PL spectroscopy, time‐resolved fluorescence (TR‐PL) spectroscopy, and photoelectrochemical measurements. The charge transfer processes of the 0.2CBB and pure CBB were investigated by PL experiments excited at 325 nm (Figure S12, Supporting Information). The fluorescence intensity of the 0.2CBB was found lower than that of the bare CBB, indicating a substantial reduction in photogenerated charge recombination.^[^ ^31^ ^]^ Furthermore, the negligible difference in TR‐PL strength of CBB was observed under the external MF (200 mT). The fluorescence lifetime obtained by fitting the TR‐PL spectrum is summarized based on Table S3 (Supporting Information) for CBB (τ = 5.10 ns), CBB+MF (τ = 5.55 ns), 0.2CBB (τ = 5.83 ns), and 0.2CBB +MF (τ = 6.68 ns) also indicates the effective prohibition of the h^+^‐e^−^ pairs recombination not only by doping but also under MF, leading to the enhanced photocatalytic efficiency (**Figure** **6**a).^[^ ^32^ ^]^

Moreover, the weak photocurrent signals were observed for CBB under light illumination, however, the obvious photocurrent signals were detected for 0.2CBB samples even without MF application. In parallel, after applying MF of 200 mT to the photoelectrode of 0.2CBB, the photocurrent density was found to be increased by 4 times for 0.2CBB as compared to the pure CBB (Figure 6b). Furthermore, the Nyquist plot of the electrochemical impedance spectrum (EIS) of obtained samples, the Nyquist plots also revealed that the Co incorporation significantly reduced the impedance effect (Figure 6c), where the effect of MF intensity on bare CBB was negligible, but the 0.2CBB showed a significant great difference. In addition, the charge transfer of the as‐prepared samples was explored by linear scanning voltammetry (LSV) and electrochemically active surface area. Under external MF (200 mT), 0.2CBB (−0.48 V) displayed the lowest overpotential than that of CBB (−0.62 V), indicating the fastest charge separation and transfer (Figure S13, Supporting Information). The findings further displayed that in both Co doping and the application of MF, the Zeeman effect prolongs the carrier lifetime, increased the photogenerated charge numbers, weakens the carrier recombination, and accelerates the electron‐hole pair transfer.^[^ ^33^ ^]^ Based on the calculations of cyclic voltammetry (CV, scanning rates from 20 to 80 mV⋅s^−1^, Figure S14, Supporting Information), the electrochemical double‐layer capacitance (C_dl_) of 0.2CBB and CBB were found to be 0.152 and 0.041 mF/cm^−2^, respectively, indicating the presence of more active sited for 0.2CBB (Figure 6d).^[^ ^34^ ^]^

In addition to charge transfer and separation dynamics, the surface reactions, i.e., adsorption and activation of CO_2_ molecules, are pivotal steps in the photoreduction of CO_2_.^[^ ^35^ ^]^ Therefore, the CO_2_ adsorption behaviours of Co‐doping at 298 K were tested (Figure S15a, Supporting Information). It was found that the 0.2CBB exhibited stronger CO_2_ uptake than pristine CBB. The Co‐doping is likely to enrich the concentration of CO_2_ around active perovskite sites to enhance CO_2_ activation and conversion. Figure S15b (Supporting Information) depicts the CO_2_ temperature‐programmed desorption (CO_2_‐TPD) spectra for Co‐doping CBB. The 0.2CBB exhibited a higher peak area under the desorption curve compared to pristine CBB, indicating a higher CO_2_ adsorption capacity. This enhanced adsorption capacity indicates that the interaction between 0.2CBB and CO_2_ is stronger, which can effectively lower the activation energy barrier and thereby promote the subsequent CO_2_ reduction process.

**Figure 7:**
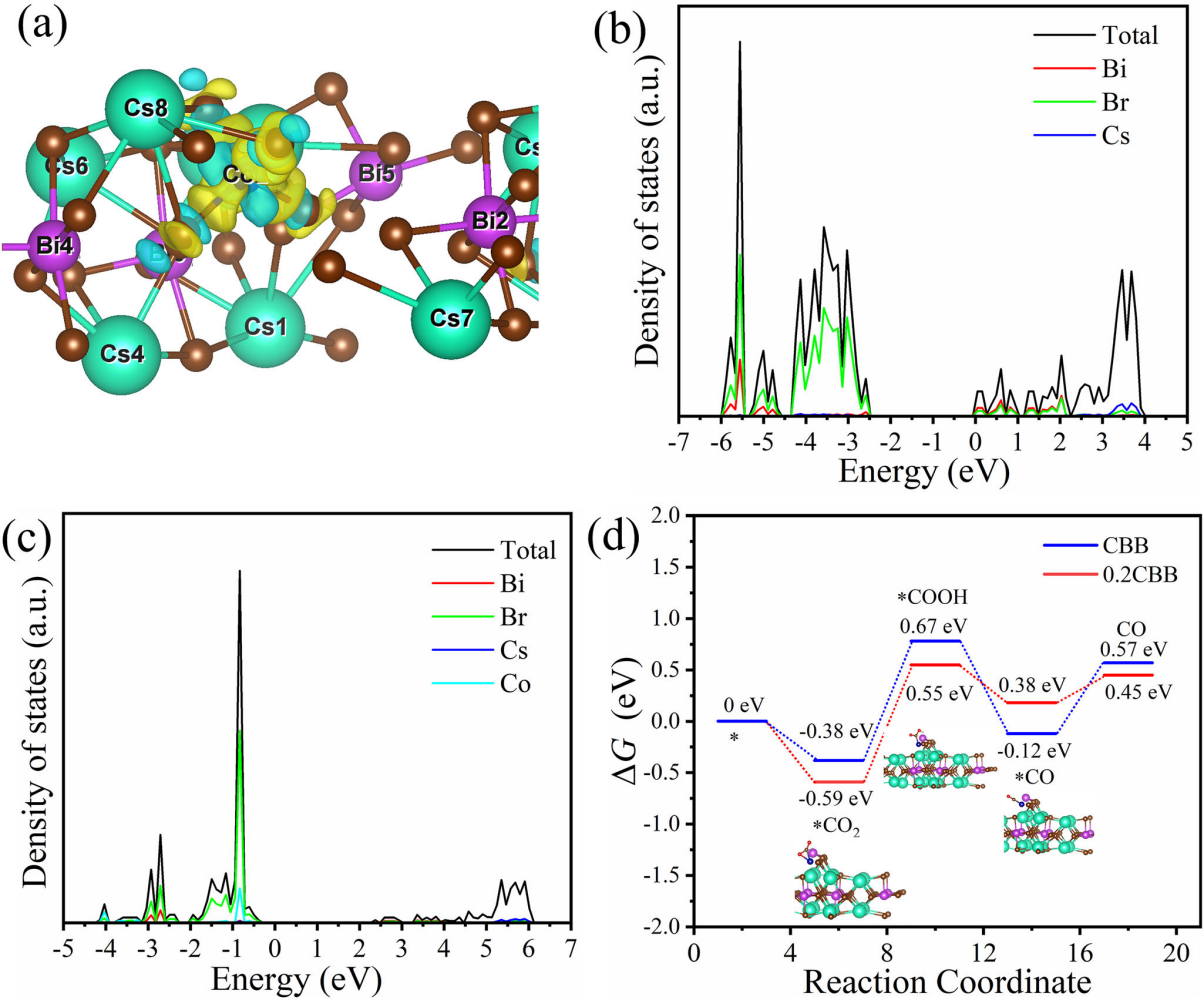
a) Charge density difference of 0.2CBB (yellow and cyan regions represent electron accumulation and depletion, respectively); Density of states (DOS) of b) CBB and c) 0.2CBB; d) Calculated Gibbs free energy diagrams for CBB and 0.2CBB.

DFT calculations were employed to investigate the spin‐state regulation effects in CO_2_ photoreduction over 0.2CBB, analyzing differential charge density, DOS, and CO_2_ adsorption energy. **Figure** **7**a shows that Co doping induced the electron localization around Co sites, facilitating reactant activation, while the spin‐polarized electrons in 0.2CBB generate spin‐polarized bands, enhancing CO_2_ adsorption at Co active sites.^[^ ^36^ ^]^ Moreover, the conduction band also confirmed the Bi 6p and Co 3d orbitals as catalytic centers (Figure 7b,c). The *d–p* orbital coupling between Co and Bi not only lowers the ^*^COOH formation barrier, but also enhances the CO production selectivity and efficiency. In addition, the Gibbs free energy analysis also revealed that the 0.2CBB has stronger CO_2_ adsorption (ΔG = −0.59 eV vs CBB's −0.38 eV) as compared to the pure CBB (Figure 7d).^[^ ^37^ ^]^ The conversion process of ^*^CO_2_ to ^*^COOH showed a lower barrier on 0.2CBB (0.55 vs 0.67 eV), while the ^*^CO desorption becomes a rate‐limiting process (ΔG = −0.12 eV vs CBB's 0.38 eV), where the subsequent protonation proceeds the CH_4_ production through favorable pathways.^[^ ^38^ ^]^

## 4 Conclusion

In summary, this study demonstrates the enhanced spin‐polarization and spin‐selective electron transfer for efficient CO_2_ photoreduction to CO through Co‐doped CBB under an external MF. The optimized 0.2CBB catalyst achieved remarkable CO and CH_4_ evolution rates of 33.04 and 10.81 µmol g^−1^ h^−1^, respectively, attributed to the Co‐induced effective CO_2_ adsorption at active sites. Under MF, the performance further improved up to 86.56 µmol g^−1^ h^−1^ (CO) and 25.36 µmol g^−1^ h^−1^ (CH_4_), representing 2.62‐ and 2.34‐fold enhancements, respectively. This marked enhancement was mainly credited to the spin‐polarized band splitting that creates selective electron transfer channels, enabling rapid exciton dissociation and directional charge transport. Moreover, DFT calculations also confirmed the Co sites as dominant catalytic centers, exhibiting low reaction energy barriers and strong electron interactions. These findings highlight the crucial role of spin polarization in CO_2_ photoconversion and provide a novel strategy for designing high‐performance catalysts with potential applications for energy and environmental issues.

## Conflict of Interest

The authors declare no conflict of interest.

## Author Contributions

F.W. contributed to formal analysis and writing—review and editing. Y.X. performed formal analysis, investigation, data collection, validation, and writing—original draft, review, and editing. Z.H. contributed to conceptualization, formal analysis, and writing—review and editing. G.H., J.Z., G.C., Y.X., and G.D. performed formal analysis. J.S. and X.F. contributed to formal analysis and writing—review and editing. M.S.S. contributed to formal analysis and editing.

## Supporting information



Supporting Information

## Data Availability

The data that support the findings of this study are available from the corresponding author upon reasonable request.
