# Supplementary material for: Manipulating the Spin State of Perovskite Cs3Bi2Br9 by Co‐Doped for Efficient Photocatalytic CO2 Reduction
Source: Adv Sci (Weinh). 2025 Nov 20;13(3):e11617. doi: 10.1002/advs.202511617 (PMC12806239; doi:10.1002/advs.202511617)
Supplement: Supplementary file 1 — Supporting Information [file ADVS-13-e11617-s001.docx]

**Manipulating the spin state of perovskite Cs_3_Bi_2_Br_9_ by Co-doped for efficient photocatalytic CO_2_ reduction**

Fahui Wang^a^, Yongmei Xia^a,c^*, Zuming He^a,f^*, Gang He^a^, Juan Zhang^a^, Jiangbin Su^a^, Guihua Chen^b^, Xiaofei Fu^c^, Muhammad Saboor Siddique^d^, Yu Xie^g^, and Guoliang Dai^e^*

*^a^Jiangxi Provincial Key Laboratory of Power Batteries & Energy Storage Materials，Xinyu University, Xinyu 338004, China; School of New Energy Science & Engineering, Xinyu University, Xinyu 338004, Jiangxi, P.R. China*

*^b^School of Pharmaceutical and Chemical Engineering, Taizhou University, Jiaojiang 318000, P.R. China*

*^c^School of Resources and Environmental Engineering, Jiangsu University of Technology, Changzhou 213001, P.R. China*

*^d^Institute of Environment and Ecology, Tsinghua-Shenzhen International Graduate School, Tsinghua University, Shenzhen 518055, China*

*^e^School of Chemistry and Life Sciences, Suzhou University of Science and Technology, Suzhou 215009, PR China*

*^f^School of Microelectronics and Control Engineering, Changzhou University, Changzhou 213164, P.R. China*

*^g^School of Environmental and Chemical Engineering, Nanchang Hangkong University, Nanchan 330063, P.R. China*

1. **Materials**

Cesium bromide (CsBr, 99.0％), Bismuth(II) bromide(BiBr_3_, 99.0％) and Cobalt bromide (CoBr_2_, 99.0％) were purchased from Aladdin Chemical Reagent Co., Ltd. (Shanghai, China). Absolute ethyl alcohol, isopropanol, dimethyl sulfoxide (DMSO), 5,5-dimethyl-pyrroline n-oxide (DMPO), and N, N-dimethylformamide (DMF) were all obtained from Sinopharm Chemical Reagent Co., Ltd. (Shanghai, China). All chemicals are of analytical grade and used without further purification.

1. **Characterization**

Powder X-ray diffraction (PXRD) analyses were conducted using a Rigaku D/MAX diffractometer with Cu Kα radiation (λ = 1.5406 Å). The morphologies and microstructures of the as-synthesized samples were characterized by scanning electron microscopy (SEM) Transmission electron microscopy (TEM) images were obtained with a JEM-1400, high-resolution TEM (HR-TEM) images with a JEM-3100, and field-emission TEM (FE-TEM) images with a Talos F200X, to observe the morphologies of all prepared catalysts. The absorption properties of the photocatalysts were analyzed using a UV-2450 spectrophotometer, with BaSO_4_ serving as a reflectance standard. X-ray photoelectron spectroscopy (XPS) analyses were performed with an ARL Quant X-ray photoelectron spectrometer using Al Kα radiation (hν = 1486.6 eV). Photoluminescence spectra (PL, Hitachi F4500, Japan) were used to characterize steady-state photoluminescence (PL) and time-resolved photoluminescence (TR-PL). ICP-OES (iCAP 7400, Thermo, China) was used to determine the element composition of samples and reaction solution. The electron spin paramagnetic spectra (EPR) were obtained electronically using a JES-FA200 (Japan) spectrometer for detecting the presence of photogenerated electrons, where the photogenerated electrons were obtained by TEMPO (2,2,6,6-tetramethylpiperidine nitrogen oxide) capture.

1. **Photoelectrochemical tests**

Mott-Schottky plot measurements and electrochemical impedance spectroscopy (EIS) were performed on a Zahner Zennium electrochemical workstation. Typically, 2 mg of photocatalyst was dispersed into 2 mL of ethanol and 10 *μ*L of Nafion mixed solution. After ultrasonic uniformity, 30 *μ*L of solution was dropped onto glassy carbon and used as a working electrode. The measurements were carried out using a three-electrode system with a Pt plate as a counter electrode and Ag/AgCl as a reference electrode. A 0.1 M Na_2_SO_4_ solution was used as the electrolyte. The frequencies of the Mott-Schottky plot measurements were 1000 Hz, respectively. The EIS measurements were performed with a bias potential of -1.2 V in the dark. Photocurrent measurements were performed on a CHI 760E electrochemical workstation. Typically, 2 mg of photocatalyst was dispersed into 2 mL of ethanol and 10 *μ*L of Nafion mixed solution. The indium-tin-oxide (ITO) glass coated with 1 cm^2^ mixed solution was used as a working electrode. The measurements were carried out using a three-electrode system with a Pt plate as a counter electrode and Ag/AgCl as a reference electrode. A 0.1 M Na_2_SO_4_ solution was used as the electrolyte. A 300 W xenon lamp with a UV cut-off filter (>380 nm) was used as the light source. The photocurrent signals of the samples were recorded under a bias potential of +0.5 V. The sample interval was 0.1 s, and the running time was 200 s.

**4.** **Magnetic circular dichroism (MCD) measurement.**

MCD spectra were acquired using a JASCO J-1500 spectropolarimeter. A 500 W Xe lamp served as the light source. The light was allowed to pass through a linear polarizer and a photoelastic modulator, controlled by a 50 kHz AC bias, to alternately generate left-handed and right-handed circularly polarized light. All MCD spectra were probed under a magnetic field of ±200 mT, which was aligned either parallel or antiparallel to the direction of light propagation. Measurements were taken at a rate of 500 nm/min with a bandwidth of 10 nm.

**5. Electron paramagnetic resonance (EPR) measurements**

EPR measurements of photogenerated holes, electrons, and C_α_ radicals were performed using a Bruker A300 EPR spectrometer. For the *in situ* EPR test for photogenerated holes (h^+^),10 mg sample and 5 mg TEMPO powder were dispersed in 10 mL CH_3_CN, which was used as a spin-trapping agent by ultrasonic treatment. After that, an 80 μL mixed solution was injected into a glass capillary and subsequently sealed in a glass tube under an argon (Ar) atmosphere for EPR testing. The EPR measurements were conducted in the absence of light, followed by UV irradiation using a 100 W Xe lamp. In this manner, the strong signal of TEMPO can be observed under dark conditions. Following illumination, TEMPO was employed to trap h^+^ and generate TEMPO^+^. Thereby the hight signal intensity of TEMPO indicates a higher concentration of h^+^.

1. **Theoretical calculation**

The density functional theory (DFT) implementation in the CP2K 6.1.0 program [1] was used to optimize different spin hybrid structures. The Quickstep method was based on a hybrid Gaussian plane wave (GPW) scheme [2, 3] with a plane wave cutoff of 500 Ry. The Goedecker-Teter-Hutter (GTH) [4] pseudopotentials were applied to describe the interactions between the core electrons in the calculations. The Perdew-Burke-Ernzerhof (PBE) [5] was used to describe the exchange-correlation effects. To

illustrate van der Waals interactions, the Grimme-D3 dispersion correction was applied in all calculations. The localized double *ξ*-valence-polarized basis set (DZVP) [6] was chosen to expand the wave functions.

In static calculations, the hybrid functions of Heyd, Scuseria, and Ernzerhof (HSE06) [7] were used to get the energy of structures in the Vienna ab initio simulation package (VASP).[8] The convergence criterion for the electronic self-consistent cycle was fixed at 10^-5^ eV. Grimme’s DFT-D3 method was applied to the long-range van der Waals (vdW) interaction corrections. The cutoff energy for the plane wave basis set was set to 400 eV. The Brillouin zone was sampled with 1 × 1 × 1 Monkhorst−Pack k-mesh. To get more accurate results, implicit solvent acetonitrile was added in all calculations based on the experimental conditions by Hennig and co-workers under the name VASPsol.[9]

**Calculation for the Free Energies.**

Based on the general single site of the CO_2_ reduction (CO_2_RR) mechanism, the pathway for CO_2_ reduction of CO in the whole reaction can be written as

* + CO_2_ →*CO_2_  (1)

*CO_2_ + H^+^ + e^-^ →*COOH (2)

*COOH + H^+^ + e ^-^→*CO + H_2_O (3)

*CO →* + CO (4)

Here, an asterisk (*) represents the active sites in the 0.2CBB catalysts. To calculate the free energy changes, a standard change of Gibbs free energy at zero potential was calculated according to the following standard formula:[10]

ΔG = ΔE + ΔZPE +ΔTS (5)

where Δ*E* refers to the change of reaction energy based on DFT simulations, ΔZPE is the zero-point energy change calculated by the vibrational frequency using the finite difference method, and Δ*S* represents the entropy change for each elementary step. The

temperature in our work is set to 298.15 K.

**7. In-situ Fourier transform infrared spectroscopy**

In-situ Fourier transform infrared spectroscopy (in-situ FT-IR) spectroscopy was conducted on a Nicolet iS50 spectrometer (Waltham, USA). The FT-IR cell was equipped with ZnSe windows. The infrared spectrum was measured with scan of 32 and aresolution of 4 cm^–1^ in the range of 400–800 cm^–1^. Prior to the tests, the catalyst was pretreated in 50mL·min^–1^ He (500 °C, 1h), and then naturally cooled to 300 °C. The background spectrumwas collected at 300, 400, and 500 °C during the cooling process and was subtracted from the sample spectra collected at the corresponding temperature. Thereafter, 2000 ppm N_2_O/He (50mL·min^–1^) was introduced into the reaction cell, and the spectra were recorded for 30min at 300, 400, and 500 °C, respectively.





**Figure.S1** SEM image of 0.2 CBB sample


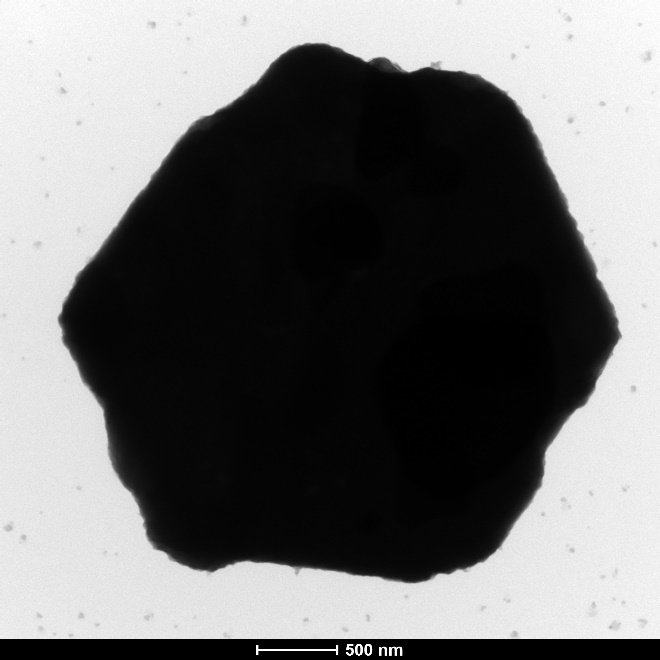


**Figure.S2** TEM image of 0.2 CBB sample


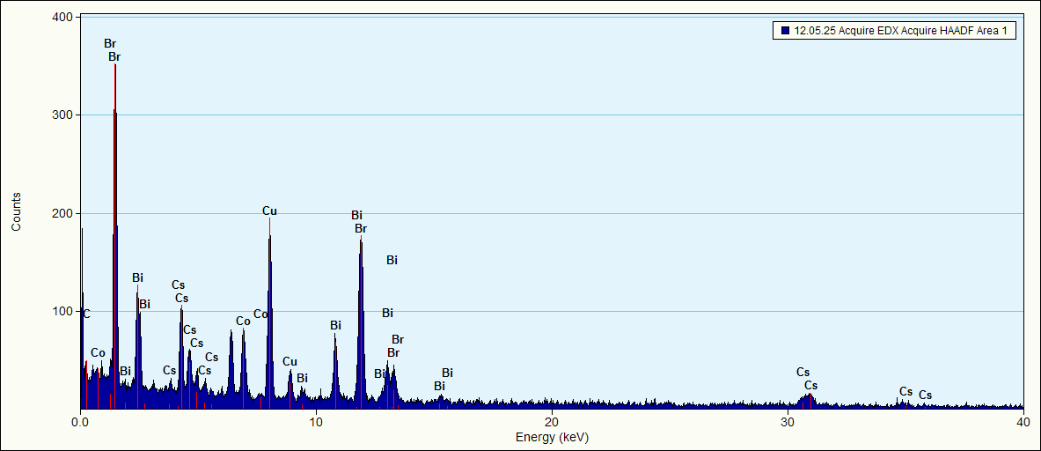


**Figure.S3** EDX image of 0.2 CBB





**Figure. S4** The magnified detail XRD patterns of the samples.





**Figure. S5** XPS survey spectra CBB and 0.2CBB of samples.





**Figure. S6** High-resolution XPS spectra Br 3d of CBB and 0.2CBB





**Figure. S7** Mott-Schottky plots for CBB and 0.2CBB samples.


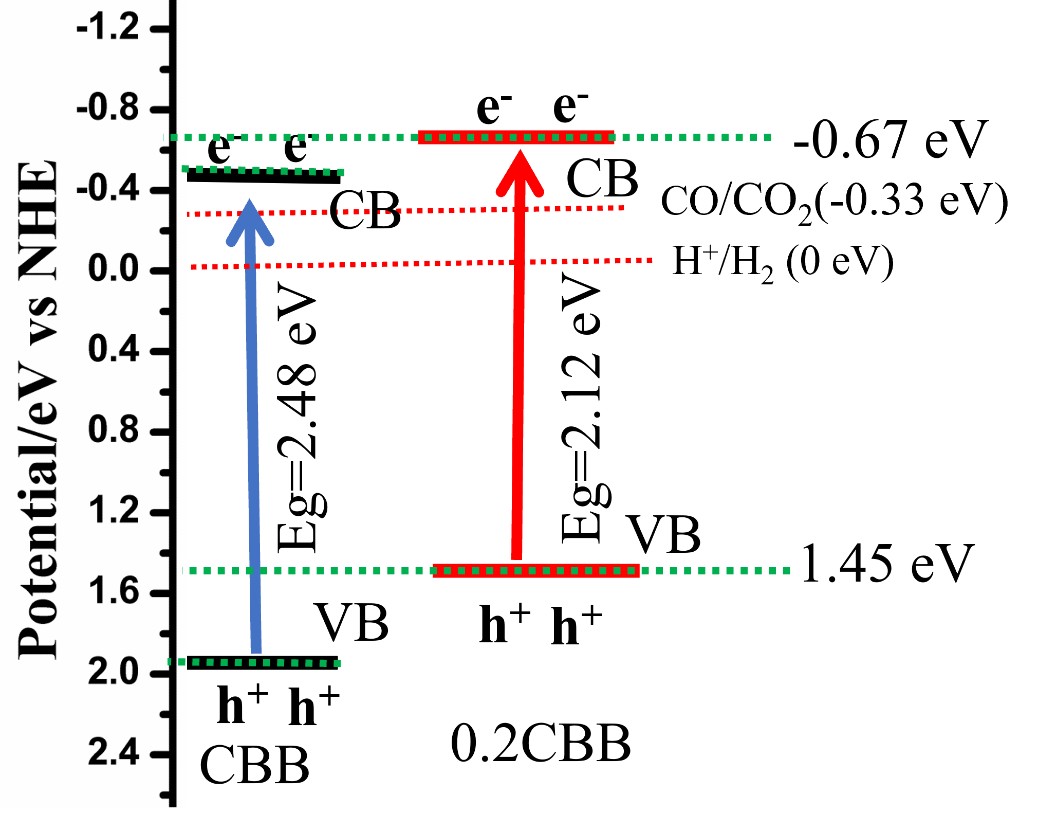


**Figure. S8** Energy band structures of 0.2CBB and CBB.





**Figure. S9** Control experiments over 0.2CBB under different conditions: light with CO_2_, dark with CO_2_, light with N_2_, and without photocatalyst.

**
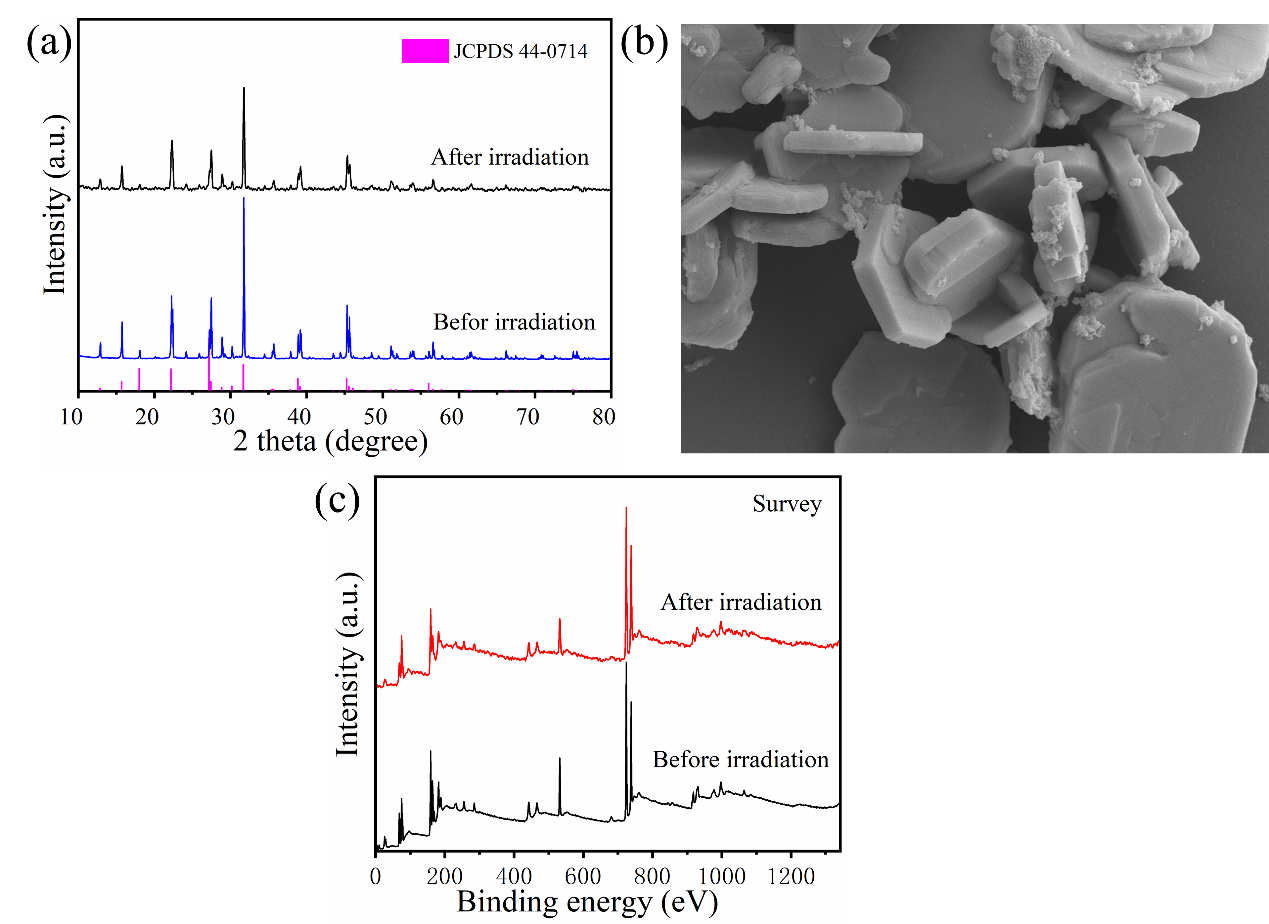
**

**Figure. S10** XRD, TEM, and XPS still maintained structural integrity both macroscopically and microscopically





**Figure. S11** The spin-polarized DOS of CBB.





**Figure.S12** PL spectra performed on the prepared photocatalysts.





**Figure.S13** LSV curves obtained in 0.1 mol L^-1^ H_2_SO_4_ electrolyte solution


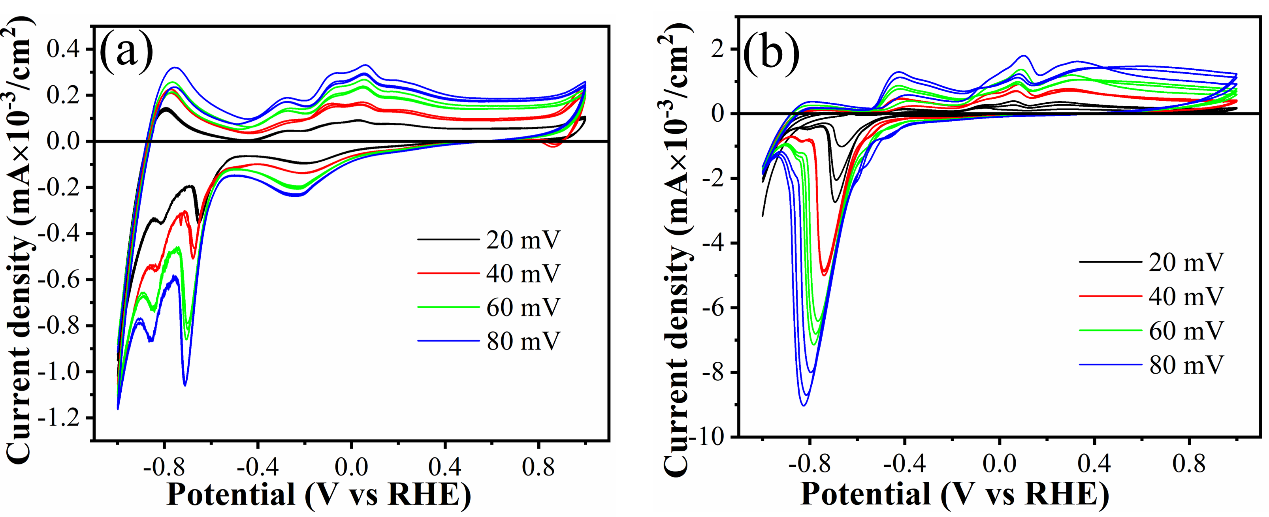


**Figure.S14** Cyclic voltammograms for measuring electrochemical double-layer capacitance (*C*_dl_) of (a) 0.2CBB and (b) CBB at different scan rates (20, 40, 60, and 80 mVs^-1^).


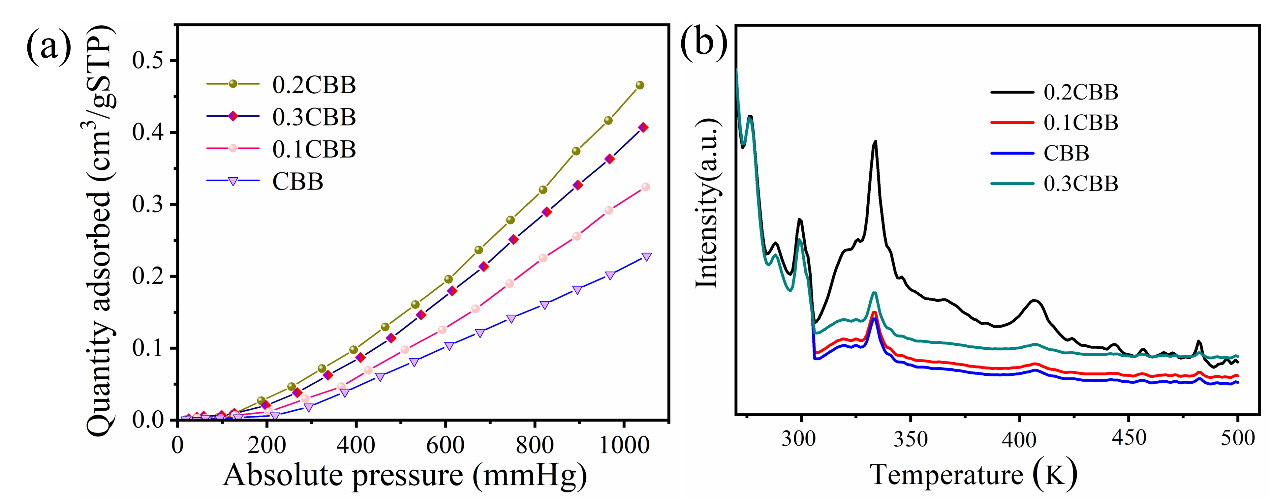


**Figure.S15** (a) CO_2_ adsorption isotherm and (b) CO_2_-TPD spectra of CBB, 0.1CBB, 0.2CBB, and 0.3CBB.

**Table S1**. **ICP-OES characterization of pristine CBB and Co-doped CBB.**

| Sample | Cs | | Bi | | Co | | Br | |
| --- | --- | --- | --- | --- | --- | --- | --- | --- |
|  | atomic | mass | atomic | mass | atomic | mass | atomic | mass |
|  | ratio | ratio | ratio | ratio | ratio | ratio | ratio | ratio |
|  | (at％) | (wt％) | (at％) | (wt％) | (at％) | (wt％) | (at％) | (wt％) |
| CBB | 21.42 | 43.62 | 14.28 | 45.73 | 0 | 0 | 64.29 | 10.64 |
| 0.1CBB | 21.42 | 44.35 | 13.57 | 44.169 | 0.72 | 0.656 | 64.29 | 10.82 |
| 0.2CBB | 21.42 | 45.1 | 12.85 | 42.55 | 1.42 | 1.33 | 64.29 | 11.0 |
| 0.3CBB | 21.42 | 45.88 | 12.15 | 40.88 | 2.12 | 2.04 | 64.29 | 11.20 |

## **Table S2. Comparison of CO_2_ photoreduction performance.**

| Catalyst | Reductant  Cocatalyst | Reaction condition | CO  (μmol h^-1^g^-1^) | Selectivity  ofCO (%) | Ref. |
| --- | --- | --- | --- | --- | --- |
| CuInS_2_/In_2_S_3_  nanotubes | H_2_O/TEOA/  Co(bpy)_3_^2+^ | 300 W Xe lamp (>380 nm) | 19.00 | / | [11] |
| CuO-TiO_2_ | H_2_O | 300 W Xe lamp | 14.54 | 63.71 | [12] |
| CuS/Pt | Na_2_SO_3_ /H_2_O | 300 W Xe lamp | 3.02 | 85.31 | [13] |
| Ni-BaTiO_3_ nanofibers | H_2_O | 300 W Xe lamp +MF | 86.47 | 65 | [14] |
| CBB/Bi_2_S_3_ | H_2_O | 300 W Xe lamp UV-Vis-NIR | 153.8 | 79.0 | [15] |
| Fe-doped CsPbBr_3_ | H_2_O/ Na_2_SO_3_ | 300 W Xe lamp (>380 nm) | 133.04 | / | [16] |
| SnO_2_/CBB | H_2_O | 300 W Xe lamp (>380 nm) | 73.6 | 70 | [17] |
| CBB/Co_3_O_4_ | H_2_O | Full spectrum, no extra heat | 168.56 | / | [18] |
| 0.2CBB | H_2_O | 300 W Xe lamp (>380 nm) +MF | 86.56 | 79.8 | **This work** |

**Table. S3.** Parameters of the TR-PL decay curves.

| **Samples** | ***τ*_1_ (ns)** | ***A*_1_** | ***τ*_2_ (ns)** | ***A_2_*** | ***τ*_ave_ (ns)*** |
| --- | --- | --- | --- | --- | --- |
| CBB | 1.23 | 913.40 | 9.16 | 123.52 | 5.20 |
| CBB+MF | 1.13 | 912.50 | 9.43 | 124.92 | 5.55 |
| 0.2CBB | 1.42 | 902.50 | 10.36 | 120.82 | 5.83 |
| 0.2CBB+MF | 1.82 | 901.03 | 12.19 | 118.68 | 6.68 |

**τ*_ave_ = (*A*_1_·*τ*_1_^2^ + *A*_2_·*τ*_2_^2^)/(*A*_1_·*τ*_1_ + *A*_2_·*τ*_2_), where *τ*_1_ and *τ*_2_ are the decay times, *A*_1_ and *A*_2_ are the PL aptitudes.

**References**

[1] Kühne, T. D.; Iannuzzi, M.; Del Ben, M.; Rybkin, V. V.; Seewald, P.; Stein, F.; Laino, T.; Khaliullin, R. Z.; Schütt, O.; Schiffmann, F.; Golze, D.; Wilhelm, J.; Chulkov, S.; Bani-Hashemian,

M. H.; Weber, V.; Borŝtnik, U.; Taillefumier, M.; Jakobovits, A. S.; Lazzaro, A.; Pabst, H.; Müller, T.; Schade, R.; Guidon, M.; Andermatt, S.; Holmberg, N.; Schenter, G. K.; Hehn, A.; Bussy, A.;

Belleflamme, F.; Tabacchi, G.; Glos, A.; Lass, M.; Bethune, I.; Mundy, C. J.; Plessl, C.; Watkins, M.; VandeVondele, J.; Krack, M.; Hutter, J. CP2K: An electronic structure and molecular dynamics software package - Quickstep: Efficient and accurate electronic structure calculations. *J. Chem. Phys.* 2020, *152*, 194103.

[2]VandeVondele, J.; Krack, M.; Mohamed, F.; Parrinello, M.; Chassaing, T.; Hutter, J. Quickstep: Fast and accurate density functional calculations using a mixed Gaussian and plane waves approach. *Comput. Phys. Commun.* 2005, *167*, 103−128.

[3] Lippert, B. G.; Parrinello, J. H.; Michele. A hybrid Gaussian and plane wave density functional scheme. *Mol. Phys.* 1997, *92*, 477−488.

[4] VandeVondele, J.; Hutter, J. Gaussian basis sets for accurate calculations on molecular systems in gas and condensed phases. *J.* *Chem. Phys.* 2007, *127*, 114105.

[5] Perdew, J. P.; Burke, K.; Ernzerhof, M. Generalized gradient approximation made simple. *Phys. Rev. Lett.* 1997, *78*, 1396−1396.

[6] Hartwigsen, C.; Goedecker, S.; Hutter, J. Relativistic separable dual-space Gaussian pseudopotentials from H to Rn. *Phys. Rev. B* 1998, *58*, 3641−3662.

[7] Krukau, A. V.; Vydrov, O. A.; Izmaylov, A. F.; Scuseria, G. E. Influence of the exchange screening parameter on the performance of screened hybrid functionals. *J. Chem. Phys.* 2006, *125*, 224106.

[8] Kresse, G.; Furthmüller, J. Efficient iterative schemes for abinitio total-energy calculations using a plane-wave basis set. *Comput.* *Mater. Sci.* 1996, *6*, 15−50.

[9] Mathew, K.; Sundararaman, R.; Letchworth-Weaver, K.; Arias, T. A.; Hennig, R. G. Implicit solvation model for density-functional study of nanocrystal surfaces and reaction pathways. *J. Chem. Phys.* 2014, *140*, No. 084106.

[10] No̷rskov, J. K.; Rossmeisl, J.; Logadottir, A.; Lindqvist, L.; Kitchin, J. R.; Bligaard, T.; Jonsson, H. Origin of the overpotential for oxygen reduction at a fuel-cell cathode. *J. Phys. Chem. B* 2004, *108*, 17886−17892

[11] Yang, J.; Zhu, X.; Mo, Z.; Yi, J.; Yan, J.; Deng, J.; Xu, Y.; She, Y.; Qian, J.; Xu, H.; Li, H. *Inorg. Chem. Front.* 2018, *5*, 3163–3163.
[12] Wang, W.; An, W.; Ramalingam, B.; Mukherjee, S.; Niedzwiedzki, D.; Gangopadhyay, S.; Biswas, P. *J. Am. Chem. Soc.* 2012, *134*, 11276–11276.
[13] Manzi, A.; Simon, T.; Sonnleitner, C.; Döblinger, M.; Wyrwich, R.; Stern, O.; Stolarczyk, J. K.; Feldmann, J. *J. Am. Chem. Soc.* 2015, *137*, 14007–14007.
[14] Xia, S. H.; Yin, X.; Chen, Y. H.; Zhang, L.; Yu, J. Y.; Ding, B.; Yan, J. H. Zeeman effect-boosted spin-polarized band splitting in diluted magnetic photocatalysis semiconductors for efficient CO₂ photoreduction. *ACS Nano* 2025, *19*(1), 1519–1529.
[15] Zhang, Z. J.; Li, H.; Wang, X. S.; Su, S. W.; Xu, J. Y. S-scheme charge transfer and photoinduced self-heating effect synergistically enhance the solar-driven CO₂ reduction over Cs₃Bi₂Br₉/Bi₂S₃ hybrid. *Chem. Eng. J.* 2024, *493*, 152473.
[16] Kim, T. H.; Cho, K.; Lee, S. H.; Kang, J. H.; Park, H. B.; Park, J. H.; Kim, Y. H. Spin polarization in Fe-doped CsPbBr₃ perovskite nanocrystals for enhancing photocatalytic CO₂ reduction. *Chem. Eng. J.* 2024, *492*, 152095.
[17] Hu, P. Y.; Liang, G. J.; Zhu, B. C.; Macyk, W.; Yu, J. G.; Xu, F. Y. Highly selective photoconversion of CO₂ to CH₄ over SnO₂/Cs₃Bi₂Br₉ heterojunctions assisted by S-scheme charge separation. *ACS Catal.* 2023, *13*, 12623–12633.
[18] Zhang, Z. J.; Qian, J. Y.; Wang, X. S.; Chu, Y. Q.; Xu, J. Y. A three-in-one integrated Cs₃Bi₂Br₉@Co₃O₄ heterostructure with photoinduced self-heating effect for synergistically enhancing the photothermal CO₂ reduction. *Small* 2024, *20*, 2401601.
